# Early neuromotor performance and later cognition in children born preterm

**DOI:** 10.1111/dmcn.14901

**Published:** 2021-05-06

**Authors:** Arend F Bos

**Affiliations:** ^1^ University Medical Center Groningen – Beatrix Children’s Hospital Division of Neonatology University of Groningen Groningen the Netherlands

## Abstract

This commentary is on the original articles by Romeo et al. and Uusitalo et al. on pages 939–946 and 947–953 of this issue.

Prediction of neurodevelopmental outcomes after preterm birth has interested neonatologists and health care workers involved in follow‐up of children born preterm for decades. Whereas many studies demonstrate evidence regarding prediction of childhood outcomes in the motor domain, prediction of cognitive skills lags behind. Two new studies add to this evidence by reporting moderately strong associations between the scores on the Hammersmith Infant Neurological Examination (HINE) in infancy and cognition later on.^1^, ^2^ It is interesting that they report similar results, with the associations remaining similarly strong after excluding children with cerebral palsy (CP) from their analyses. The findings in both studies are robust and include the complete range of preterm birth. They encompass the HINE performed at 3 to 12 months^1^ and at 24 months,^2^ with cognitive development measured at 2 years^1^ and 11 years^2^ respectively.

The associations found have also been demonstrated in several studies regarding the predictive value of General Movement Assessment (GMA) for later cognition.^3^ Both HINE and GMA have good predictive value for CP, but associations with cognition are only moderately strong. Both instruments thus enable us to identify risk of cognitive delay at group level, but there is considerable overlap of scores between children with typical and delayed cognitive development. This may preclude prediction on an individual basis, as sensitivities between 3 to 12 months vary from 50% to 80%. It would be interesting to investigate whether the combination of GMA and HINE in young infants would increase the predictive value for later cognition, in line with what has been found for prediction of CP.

The connection between early findings in the motor domain and later cognitive skills is very interesting. In typically developing children born at term, motor development stimulates interaction with the environment, resulting in advancing development of various cognitive skills, by creating new learning opportunities.^4^ Studies in children born preterm are scarce, but we can extrapolate from these findings. Although perinatal adverse events may disrupt neuronal structures and connections in the developing brain, affecting both motor and cognitive brain areas, an alternative hypothesis is that certain specific movements and postural patterns during infancy are required for the infant to develop cognitive skills. If these patterns are atypical, abnormal, or absent, cognitive development will be impaired, because of disruptions of brain areas involved in motor skills, rather than cognition.^4^ This could also explain the lack of clear association between magnetic resonance imaging findings and later cognition in children born preterm.

The cause of the coocurring motor and cognitive delay could be different between children born early preterm and moderate‐to‐late preterm. With advancing gestational age, the disruptions of brain development are less severe, but the associations are still moderately strong.^1^ In line with this, it has been reported that, in children born moderate‐to‐late preterm, earlier attainment of walking is associated with good problem‐solving skills at 4 years.^5^ But even in children born very preterm, cognitive development may be based on acquiring early motor skills. In the study by Uusitalo et al., the HINE (at 2y) and cognitive scores (at 11y) were associated 9 years apart, whilst this association was not found for impaired motor outcomes such as developmental coordination disorder.^2^

The findings in both studies have important clinical implications. Lower scores on the HINE may identify children born preterm at risk for cognitive delay, enabling interventions aimed at improving later cognition to start earlier than at present. It is tempting to conceive interventions that improve certain movements and postures because these were the items of the HINE that were associated most strongly with attainment of cognitive skills. Whether this indeed improves cognition in children born preterm should be the subject of future longitudinal studies.
